# When Modern Technology Meets Ancient Traditional Chinese Medicine

**DOI:** 10.1155/2015/156581

**Published:** 2015-02-19

**Authors:** Calvin Yu-Chian Chen, James David Adams, Tingjun Hou, Gerhard Litscher

**Affiliations:** ^1^Human Genetic Center, Department of Medical Research, China Medical University Hospital, Taichung 40447, Taiwan; ^2^Research Center for Chinese Medicine & Acupuncture, China Medical University, Taichung 40402, Taiwan; ^3^School of Medicine, College of Medicine, China Medical University, Taichung 40402, Taiwan; ^4^Department of Biomedical Informatics, Asia University, Taichung 41354, Taiwan; ^5^Department of Pharmacology and Pharmaceutical Sciences, University of Southern California, School of Pharmacy, Los Angeles, CA, USA; ^6^College of Pharmaceutical Sciences, Zhejiang University, Hangzhou, Zhejiang 310058, China; ^7^TCM Research Center Graz, Medical University of Graz, 8036 Graz, Austria

In this issue, we select several papers related to how modern technology modernizes ancient traditional Chinese medicine such as acupuncture, herbal medicine, and other alternative treatments. There are 9 papers collected in this special issue. In this issue, we particularly picked some research articles which use computational techniques to approach ancient traditional Chinese medicine (TCM). No doubt TCM has a very strong clinical and experience base lasting for thousands of years. However, TCM is still a complementary and alternative medicine (CAM) because of its mysterious theoretical basis, including qi and blood. However, there are plenty of investigations that have identified the efficiency of TCM and acupuncture. In this issue, we present the issue of peroxisome proliferator-activated receptors (PPARs), acute lymphoblastic leukemia (ALL), protein phosphatase 2A (PP2A), diabetic nephropathy, antihepatic steatosis components within Coptidis Rhizoma alkaloids extract (CAE), dermal microcirculation blood perfusion characterization of meridian channels (acupoints), acute spinal cord injury, and nucleoside analogues (NAs) and a review paper of cerebrospinal fluid pharmacology.

The paper by K.-C. Chen and C. Y.-C. Chen raises an interesting issue about PPARs related to regulation of lipid metabolism, inflammation, cell proliferation, differentiation, and glucose homeostasis and control of related ligand-dependent transcription networks of genes. They screen potent compounds from TCM databases. Biological activity prediction using multiple linear regression (MLR), support vector machines (SVM), and Bayes network toolbox (BNT) models is utilized in this paper for more evidence of these potent TCM compounds. Furthermore, a molecular dynamics simulation is performed with a high speed workstation. The investigation shows that these TCMs might be potent leads for PPARs. Another paper contributed by Y.-L. Hsiao et al. uses different approach and method. They screen potent leads from Shanghai Innovative Research Center of Traditional Chinese Medicine (http://www.sirc-tcm.sh.cn/en/index.html) and use the MLR model and SVM model for further investigation. More evidence is also shown with CoMFA and CoMSIA models. Protein phosphatase 2A (PP2A) is an important phosphatase which regulates various cellular processes, such as protein synthesis, cell growth, cellular signalling, apoptosis, metabolism, and stress responses. K.-C. Chen et al. discover few potent TCM compounds from TCM databases by screening more than 61000 TCM compound databases. The disordered part of a protein might influence the efficacy of a drug; thus they analyse the disordered part in the first stage. It is a very new and important concept for screening and docking. However, in most of the recent studies, it seems that it is still not in practice yet. Their Figure 1 indicates that the structure of the binding domain is stable as the major residues of the binding domain do not lie in the disordered region. This study also performs a time-consuming molecular dynamics experiment for further clarity.

L. Zhang et al. show their ability in text mining of the classical medical literature for medicines that show potential in diabetic nephropathy. They mention in the conclusion, “The methods developed in this study offer a targeted approach to identifying traditional herbs and/or formulae as candidates for further investigation in the search for new drugs for modern disease. However, more effort is still required to improve our techniques, especially with regard to compound formulae.” H. Fan et al. publish a paper entitled “*In Vitro Screening for Antihepatic Steatosis Active Components within Coptidis Rhizoma Alkaloids Extract Using Liver Cell Extraction with HPLC Analysis and a Free Fatty Acid-Induced Hepatic Steatosis HepG2 Cell Assay*” and show significant results, finding two potent and active compounds within CAE. This indicates that the screening method they developed is a feasible, rapid, and useful tool for studying TCMs in treating hepatic steatosis.

Y. Wu et al. contribute a significant review article entitled “*Cerebrospinal Fluid Pharmacology: An Improved Pharmacology Approach for Chinese Herbal Medicine Research.*” They make an important conclusion we quote here, “In summary, CSFP provides a new strategy not only to eliminate some barriers of CHM research for treating ND, but also to broaden the pharmacology research for bridging the gap between CHM and modern medicine. Moreover, the advancements in CSFP will bring about a conceptual move in active ingredients discovery of CHM and make a significant contribution to CHM modernization and globalization.”

D. Zhou et al. contribute a research article entitled “*Microcirculation Perfusion Monitor on the Back of the Health Volunteers.*” They observe some of the characteristics of the dermal microcirculation blood perfusion of the governor meridian. All of the features of the governor meridian have not been shown. In future research, they plan to accomplish the characterization of all of the features of the governor meridian as well as the other twelve regular meridians and draw the specific dermal microcirculation blood perfusion graphs of the fourteen meridians in order to provide evidence that microcirculation changes after interventions or under pathological conditions.

M. Du et al. publish an interesting paper entitled “*A Brief Analysis of Traditional Chinese Medical Elongated Needle Therapy on Acute Spinal Cord Injury and Its Mechanism.*” From their investigation, we learn that elongated needle therapy has an obvious effect on acute spinal cord injury in rabbits. Its mechanism involves inhibiting the expression of the Fas → caspase-3 cascade, thereby inhibiting cell apoptosis after spinal cord injury.

L. Min et al. show their powerful ability in mathematic modelling investigation in TCM. We quote their important results, “The modelling analysis with the experimental data analysis motivates to propose the previous three hypotheses, which may interpret some clinical experience judgements. The dynamics of anti-HBV infection therapy are very complex. It is difficult to set up mathematical model to describe them accurately. However, modelling dynamics of anti-HBV infection therapy would enable a better understanding, prediction, and design of anti-HBV infection treatments.”

Overall, we can see that modern technology is used for investigating TCM related studies. We also hope more modern techniques can be applied in TCM studies to speed up the modernization of TCM.

